# ﻿A checklist of black flies (Diptera, Simuliidae) from India

**DOI:** 10.3897/zookeys.1118.84686

**Published:** 2022-08-24

**Authors:** Arka Mukherjee, Atanu Naskar, Oishik Kar, Debdeep Pramanik, Dhriti Banerjee

**Affiliations:** 1 Zoological Survey of India, Prani Vigyan Bhawan, M-Block, New Alipore, Kolkata, 700053, West Bengal, India Zoological Survey of India Kolkata India

**Keywords:** Diversity, inventory, onchocerciasis, public health

## Abstract

An updated checklist of the family Simuliidae from India is presented. A total of 79 species of *Simulium* belonging to eight different subgenera are listed. Eleven species that were not reported in the previous checklist are added here. The present list contributes to a better understanding of the diversity of Simuliidae in India, as well as the impact of *Simulium* species on the public health of this mega-diverse country.

## ﻿Introduction

Black flies (Diptera, Nematocera, Simuliidae) are infamous as vectors for transmitting onchocerciasis (river blindness) in many countries throughout the world (Datta 1997). However, in India, onchocerciasis is still not a serious concern despite some sporadic cases in North-east India (Barua et al. 2011). The total number of simuliid species throughout the world is estimated to be 2,415, of which 2,398 species are living and 17 are fossil species (Adler 2022). An inventory of world Simuliidae was first published by Crosskey (1988) and has since been updated almost every year. The beginning of simuliid research was in the early 19^th^ century when Latreille (1802) described *Simulium*Latreille, 1802. The family Simuliidae Newman, 1834 was established by Newman (1834) with the inclusion of only the genus *Simulium*. According to Mitra et al. (2017), the first description of *Simulium* from India was probably by Becher (1885), and since then, researchers have described new species of simuliids from the country (Brunetti 1911; Senior-White 1922a, 1922b; Edwards 1927; Puri 1932a, 1932b, 1932c, 1932d, 1932e, 1933a, 1933b, 1933c). In the second half of the 20^th^ century, the simuliid research was mainly undertaken by Datta (1973, 1974a, 1974b, 1975, 1978, 1980, 1983, 1985, 1992a, 1992b, 1997). He extensively studied the Indian simuliid fauna, described several new species of *Simulium*, and provided new distributional records. His review of the family in the Oriental realm (Datta 1983) is an exceptional piece of work in its understanding of the biology of black flies. Datta and Pal (1975); Datta et al. (1975) described a few additional rare species of the subgenus Simulium, especially from the Darjeeling area of West Bengal state, and provided taxonomic identification keys to simuliid species found in that region. Later, in the 21^st^ century, several researchers discovered many new species (Mitra et al. 2006; Takaoka and Somboon 2008; Shrestha and Takaoka 2009; Mitra and Sharma 2010; Takaoka et al. 2010, 2011; Henry et al. 2011; Borah et al. 2012; Dutta Saha et al. 2012; Anbalagan et al. 2014, 2015a, 2015b). The first checklist of Simuliidae of India was published by Mitra et al. (2017). The most recent publications are by Anbalagan et al. (2020a, 2020b) in which they described three new species of the Simulium —two in the subgenus Gomphostilbia and one in the subgenus Nevermannia.

The present work documents 79 species of Simuliidae belonging to eight subgenera of *Simulium*, based on a review of the World Catalog of Simuliidae (Adler 2022) and other literature. The binomial names are based on the valid nomenclature of Systema Dipterorum (Evenhuis and Pape 2022). Eleven species have been added here to those species already included in the earlier checklist of Indian Simuliidae by Mitra et al. (2017). The inclusion of these previously unreported species will help us to better understand the taxonomy and diversity of the black flies in India. It will also lead to the assessment of the newly reported species as potential vectors of onchocerciasis in India.

## ﻿Results

### ﻿Order Diptera Linnaeus, 1758

**Infraorder Culicomorpha Hennig, 1948**

**Superfamily Chironomoidea Newman, 1834**

**Family Simuliidae Newman, 1834**

**Subfamily Simulinae Newman, 1834**

**Tribe Simuliini Newman, 1834**

#### Genus *Simulium*Latreille, 1802

##### Subgenus Eusimulium Roubaud, 1906

1. Simulium (Eusimulium) aureum Fries, 1824

2. Simulium (Eusimulium) weiningense Chen & Zhang, 1997

##### Subgenus Gomphostilbia Enderlein, 1973

3. Simulium (Gomphostilbia) bucolicum Datta, 1975

4. Simulium (Gomphostilbia) darjeelingense Datta, 1973

5. Simulium (Gomphostilbia) fidum Datta, 1975

6. Simulium (Gomphostilbia) litoreum Datta, 1975

7. Simulium (Gomphostilbia) metatarsale Brunetti, 1911

8. Simulium (Gomphostilbia) pattoni Senior-White, 1922

9. Simulium (Gomphostilbia) sundaicum Edwards, 1934

10. Simulium (Gomphostilbia) tenuistylum Datta, 1973

11. Simulium (Gomphostilbia) unum Datta, 1975

In the revised inventory of Adler (2022), this species was documented from Java (Indonesia) and Malaysia. However, Datta (1991) reported its occurrence in the Bankura and Purulia districts (23°14'28.7"N, 87°3'42.6"E and 23°19'57.1"N, 86°21'46.8"E) in West Bengal, India. It was not included in Mitra et al.’s (2017) checklist.

12. Simulium (Gomphostilbia) agasthyamalaiense Vijayan, Anbalagan, Rekha, Dinakaran & Krishnan, 2019

This species was described by Vijayan et al. (2019) from reared pupa collected in Agasthyamalai Biosphere Reserve, Tamil Nadu (8°55'4.1"N, 77°36'1.6"E), India.

13. Simulium (Gomphostilbia) barnesi Takaoka & Suzuki, 1984

14. Simulium (Gomphostilbia) cauveryense Anbalagan, 2015

15. Simulium (Gomphostilbia) decuplum Takaoka & Davies, 1995

16. Simulium (Gomphostilbia) dinakarani Anbalagan, 2020

This species was described by Anbalagan et al. (2020a) and occurs in Tamil Nadu (10°27'30.7"N, 78°01'15.7"E), India.

17. Simulium (Gomphostilbia) kottoorense Anbalagan, 2015

18. Simulium (Gomphostilbia) krishnani Anbalagan, 2020

This species was reported from Karnataka (14°58'36"N, 74°46'40"E), India, by Anbalagan et al. (2020a).

19. Simulium (Gomphostilbia) kumbakkaraiense Anbalagan, 2019

20. Simulium (Gomphostilbia) panagudiense Anbalagan 2015

This species was recently described by Anbalagan et al. (2019) with distributional records in Tamil Nadu (10°18'30.3"N, 77°53'35.8"E), India.

21. Simulium (Gomphostilbia) parahiyangum Takaoka & Sigit, 1992

22. Simulium (Gomphostilbia) peteri Anbalagan, 2014

23. Simulium (Gomphostilbia) sachini Takaoka & Henry, 2010

24. Simulium (Gomphostilbia) takaokai Anbalagan, 2014

25. Simulium (Gomphostilbia) williei Takaoka & Thapa, 2010

##### Subgenus Montisimulium Rubstov, 1974

26. Simulium (Montisimulium) dasguptai Datta, 1974

27. Simulium (Montisimulium) dattai Takaoka & Somboon, 2008

28. Simulium (Montisimulium) ghoomense Dutta, 1973

29. Simulium (Montisimulium) nemorivagum Datta, 1973

30. Simulium (Montisimulium) yuntaiense Chen, Wen & Wei, 2006

##### Subgenus Nevermannia Enderlein, 1921

31. Simulium (Nevermannia) aureohirtum Brunetti, 1911

32. Simulium (Nevermannia) gracilis Datta, 1973

33. Simulium (Nevermannia) karavalliense Anbalagan, Rekha, Vijayan, Balachandran, Dinakaran & Krishnan, 2020

This species was reared from pupae collected in Tamil Nadu (11°20'03.8"N, 78°19'19.6"E), India and described by Anbalagan et al. (2020b).

34. Simulium (Nevermannia) praelargum Datta, 1973

35. Simulium (Nevermannia) purii Datta, 1973

36. Simulium (Nevermannia) rufithorax Brunetti, 1911

37. Simulium (Nevermannia) senile Brunetti, 1911

38. Simulium (Nevermannia) subratai Takaoka, Thapa & Henry, 2011

39. Simulium (Nevermannia) wichaii Takaoka, 2010

##### Subgenus Tetisimulium Rubtsov, 1960

40. Simulium (Tetisimulium) stevensoni Edwards, 1927

##### Subgenus Himalayum Lewis, 1973

41. Simulium (Himalayum) indicum Becher, 1885

##### Subgenus Simulium Latreillie, 1802

42. Simulium (Simulium) alajense Rubtsov, 1938

43. Simulium (Simulium) asishi Datta, 1985

44. Simulium (Simulium) adventicium Datta, 1985

45. Simulium (Simulium) barraudi Puri, 1932

46. Simulium (Simulium) biforaminiferum Datta, 1974

47. Simulium (Simulium) consimile Puri, 1932

48. Simulium (Simulium) christophersi Puri, 1932

49. Simulium (Simulium) dentatum Puri, 1932

50. Simulium (Simulium) digitatum Puri, 1932

51. Simulium (Simulium) ephemerophilum Rubstov, 1947

52. Simulium (Simulium) gravelyi Puri, 1933

53. Simulium (Simulium) griseifrons Brunetti, 1911

54. Simulium (Simulium) grisescens Brunetti, 1911

55. Simulium (Simulium) gurneyae Senior-White, 1922

56. Simulium (Simulium) himalayense Puri, 1932

57. Simulium (Simulium) hirtipannus Puri, 1932

58. Simulium (Simulium) howletti Puri, 1932

59. Simulium (Simulium) kapuri Datta, 1975

60. Simulium (Simulium) lineothorax Puri, 1932

61. Simulium (Simulium) nigrifacies Datta, 1974

62. Simulium (Simulium) nilgiricum Puri, 1932

63. Simulium (Simulium) nitidithorax Puri, 1932

64. Simulium (Simulium) novolineatum Puri, 1933

65. Simulium (Simulium) nodosum Puri, 1933

66. Simulium (Simulium) pallidum Puri, 1932

67. Simulium (Simulium) palmatum Puri, 1932

68. Simulium (Simulium) palniense Puri, 1932

69. Simulium (Simulium) pradyai Takaoka & Somboon, 2008

70. Simulium (Simulium) pothigaiense Anbalagan, 2018

71. Simulium (Simulium) ramosum Puri, 1932

72. Simulium (Simulium) rashidi Lewis, 1973

73. Simulium (Simulium) rufibasis Brunetti, 1911

74. Simulium (Simulium) singtamense Datta & Pal, 1975

75. Simulium (Simulium) striatum Brunetti, 1912

76. Simulium (Simulium) tenuitarsus Puri, 1933

77. Simulium (Simulium) valparaiense Anbalagan, 2018

78. Simulium (Simulium) yanaense Anbalagan, 2019

This species was described from Karnataka (14°31'19.2"N, 74°19'12"E), India by Anbalagan et al. (2019).

##### Subgenus Wilhelmia Enderlein, 1921

79. Simulium (Wilhelmia) pseudequinum Séguy, 1921
